# High frequency multiscale relationships among major cryptocurrencies: portfolio management implications

**DOI:** 10.1186/s40854-021-00290-w

**Published:** 2021-09-13

**Authors:** Walid Mensi, Mobeen Ur Rehman, Muhammad Shafiullah, Khamis Hamed Al-Yahyaee, Ahmet Sensoy

**Affiliations:** 1 Department of Economics and Finance, College of Economics and Political Science, Sultan Qaboos University, Muscat, Oman; 2 Shaheed Zulfikar Ali Bhutto Institute of Science and Technology (SZABIST), Islamabad, Pakistan; 3 School of Economics, University of Nottingham Malaysia, Jalan Broga, 43500 Semenyih, Selangor Malaysia; 4 Muscat University, Al Ghubrah North, Muscat, Oman; 5 Faculty of Business Administration, Bilkent University, 06800 Ankara, Turkey

**Keywords:** Cryptocurrency, High frequency analysis, Nonlinear multiscale causality, Rolling window wavelet correlation, G14

## Abstract

This paper examines the high frequency multiscale relationships and nonlinear multiscale causality between Bitcoin, Ethereum, Monero, Dash, Ripple, and Litecoin. We apply nonlinear Granger causality and rolling window wavelet correlation (RWCC) to 15 min—data. Empirical RWCC results indicate mostly positive co-movements and long-term memory between the cryptocurrencies, especially between Bitcoin, Ethereum, and Monero. The nonlinear Granger causality tests reveal dual causation between most of the cryptocurrency pairs. We advance evidence to improve portfolio risk assessment, and hedging strategies.

## Introduction

Analysis of co-movements and Granger causality across frequencies attracts a special attention in much of the contemporary theoretical and empirical research in finance with regards to analysis on contagion, volatility spillovers, predictability, bubbles, and crashes (e.g., Wang et al. 2017; Saâdaoui et al. 2017; Rehman and Apergis 2019; Bouri et al. 2019).^1^ In recent times, the finance literature has increasingly borrowed estimation techniques from physics—i.e. wavelet transformation of data to different time-scales—to analyze the multiscale relationship and directional Granger causality between assets and/or markets^2^ (Mensi et al. 2019). These analyses have important implications on diversification benefits, hedging strategies, and portfolio risk assessment.

Cryptocurrencies have exhibited spectacular growth since their inception in 2008, with the range of different currencies recently surpassing 3000. This digital money (Financial technology) reduces the transaction costs, provides higher quality services, and increases customer satisfaction (Kou et al. 2021a). Their emergence in such numbers underpins their importance to market participants, governments, firms, and economists. Interestingly, the increasing integration and interdependence among international markets reduces the diversification opportunities. In addition, cryptocurrencies have been considered a viable alternative to traditional assets, especially in the wake of the global financial crisis (GFC) of 2008 (Corbet et al. 2019; Rehman and Vo 2020). Thus, a better understanding of the multiscale interactions among major cryptocurrencies may provide new opportunities for investors.

In the literature, different econometric methods can be used to analyze the relationships among markets (causality test, cointegration, bivariate GARCH models, structural vector autoregression (SVAR), spillover index, copula functions, and quantile regression approach, among others). However, these methods are not able to examine the relationships under both time–frequency domain. Our research philosophy is to account for the evolving relationship between cryptocurrency markets not only over time but also over frequencies to account for market heterogeneity (traders and speculators are interested in short-term investment (high frequencies) and institutional investors are concerned by the long term investments (low frequencies).

This study contributes to the emerging empirical literature in three important fronts. First, it uses high frequency data to examine the multiscale interactions among main cryptocurrencies—Bitcoin, Ethereum, Litecoin, Dash, Ripple, and Monero—in terms of market capitalization and their remarkable trading volume in the last few years (Bouri et al. 2020a). The relatively large sample used in this study adds to the literature on co-movement and interconnectedness of cryptocurrencies—by providing a richer and an extensive empirical analysis. This contrasts with many earlier studies that limit their analyses to a few cryptocurrencies—see e.g., Corbet et al. (2018a), Phillip et al. (2018), Aslanidis et al. (2019) and Qureshi et al. (2020). The use of high frequency data offers an additional useful information on how crypto markets co-move and respond to local and international shocks. The high frequency data has significant power in predicting stock prices and more advantageous than low-frequency data (Zhang and Wang 2019). Koopman et al. (2005) argue that volatility models based on high-frequency estimators enable forecasts that are superior to those generated by models grounded in low-frequency data. Thus, the high frequency data researchers can set a sufficiently narrow time window around each market announcement to check if markets are surprised or not by a specific news. Measuring the surprise on this limited time horizon allows to remove the noise deriving from other events that might influence the instrument’s quote along the day and potential crowding-in or out effects. Second, it overcomes the static full sample analysis by relying on rolling window wavelet correlation (RWWC) coefficients to analyze the co-movements among cryptocurrency returns over different time scales (Polanco-Martínez et al. 2018). We notice that the rolling window analysis assesses the stability of the coefficients over time.

Despite their advantage, RWWC is unable to identify the directional causality (uni- or bi-directional causalities) and the magnitude of causalities at different frequencies. To do this, we use the non-linear directional Granger causality at multiple scale to identify the origin of information transmission. We notice that the presence of unidirectional causality from market $i$ to market $j$ indicates that market $i$ can be used to predict the price return of market $j$. In addition, the decomposition of raw return series is fundamental to account for the heterogeneous investors. Thus, we apply for a deepen analysis the nonlinear Granger causality test of Diks and Panchenko (2006) to find out the wavelet decomposition coefficients. The advantage of this nonlinear causality testing method is that, while the wavelet correlation coefficients measure the co-movements, they are unable to establish direction of causation in different wavelet scales. Determining the direction of causation allows us to determine the direction of information flow (and perhaps spillover of volatility and contagion) across different time horizons to construct a more complete picture of the cryptocurrency marketplace.

Third, the findings of RWWC and multiscale Granger causality methods are relevant for cryptocurrency investors in terms of portfolio design. For this aim, we assess an investment strategy of mixed portfolios composed from Bitcoin and each of other cryptocurrency by quantifying the optimal weights, hedge ratios and hedging effectiveness under different wavelet scales. We select Bitcoin as a benchmark because it is the largest cryptocurrency asset and widely traded and accepted by investors. From a practical perspective, considering these methods together provide an accurate and rich information to cryptocurrency investors and fund managers to enhance their funds allocation and investment trading strategies.

Our results demonstrate that the cryptocurrencies move together—largely in a positive direction—and experience long-term memory. This observation holds more strongly for the cryptocurrency pairs involving Bitcoin, Ethereum, and Monero. Bidirectional causality exists between most of the cryptocurrency pairs as per the Diks and Panchenko (2006) tests. The implication of these findings is that there exists substantial scope for arbitrage, portfolio risk assessment, diversification, and improvement (Corbet et al. 2018b; Makarov and Schoar 2019). Portfolio managers and investors who engage across investment horizons (i.e., multi-prospect and/or high-frequency traders) can avail these findings for their strategic decisions (Qureshi et al. 2020). The analysis of cryptocurrency pricing data at 15-min intervals may help the agents in managing and stabilizing their intra-day transaction portfolio to manage risk and ensure predictability of returns. Of late, the high levels of volatility exhibited in the cryptocurrency markets make them a perfect candidate for high-frequency volatility analysis. As mentioned above, high-frequency analysis provides richer and more useful insights regarding market response to specific news, noisy events, and/or shocks. The findings from this high-frequency analysis may additionally allow agents and policymakers to improve stability of the system’s connectedness network (Bouri et al. 2021a).

The remainder of this paper is structured as follows: “Literature review” section presents the succinct literature review; “Data and methodology” section explicates the materials; “Results” section discusses the empirical results; “Conclusion” section concludes the paper.

## Literature review

The literature on cryptocurrency has grown significantly in the recent years. Yaya et al. (2019) analyze the persistence and the evolving interdependence between BTC and other major cryptocurrencies and reveal significant cointegration between cryptocurrency prices and BTC and an increase in both volatility persistence and efficiency in cryptocurrency markets after the onset of the price crash in 2017/2018. Koutmos (2018) finds evidence of spillovers in returns and volatility among cryptocurrencies and that news announcement amplify the degree of spillovers. Ji et al. (2019) apply the spillover index to explore the connectedness between various cryptocurrencies and show that Bitcoin (Ethereum) is the dominant transmitter (receiver) of spillover to (from) others, supporting the findings of Corbet et al. (2018a).

Mensi et al. (2019) use wavelet approach and find that a portfolio composed from Bitcoin and other cryptocurrencies provides diversification benefits where Bitcoin-Ethereum portfolio offers the highest hedging effectiveness. Using rescaled range and wavelet method, Celeste et al. (2019) analyze the multifractal property of Bitcoin, Ethereum and Ripple price behavior. The results show that Bitcoin exhibit a long memory process and cyclical persistence and anti-persistence process. Another strand of empirical literature has addressed the relationships between cryptocurrencies under time–frequency space. Omane-Adjepong and Alagidede (2019) use wavelet approach and parametric and nonparametric causality tests to examine the spillovers and causality in returns and volatility returns between seven cryptocurrencies. Mensi et al. (2019) analyzed the co-movements between Bitcoin and five major cryptocurrencies using wavelet coherence and cross wavelet transform approaches. They find evidence of multiscale co-movements between Bitcoin and other cryptocurrencies and that a mixed cryptocurrency portfolio offers diversification benefits.

Chaudhari and Crane (2020) investigate the cross-correlations among cryptocurrencies. Ferreira et al. (2020) use the detrended cross-correlation (DCCA) and detrending moving-average cross-correlation (DMCA) correlation coefficients to examine the correlation structure among the most liquid cryptocurrencies (Bitcoin, DASH, Stellar, Litecoin, Monero, and Ripple). Nie (2020) uses the multidimensional scaling method to analyze the evolving correlations in cryptocurrency markets. Qureshi et al. (2020) avail wavelet methodologies to observe the interdependencies across five dominant as well as liquid cryptocurrencies—Bitcoin, Ethereum, Ripple, Litecoin, and Bitcoin Cash)—to alternate between time and frequency. Mensi et al. (2020) and Rehman et al. (2020) examine the diversification properties of bitcoin with Islamic asset classes and highlight significant spillover effect from Bitcoin to Islamic stocks. Bouri et al. (2020a) observe the ‘volatility surprise’ of major cryptocurrencies over frequency domain and find that causality is determined by permanent shocks over the short horizon and by transitory shocks over the long horizon. The authors conclude that Bitcoin is not the only dominant cryptocurrency in the market. Bouri et al. (2020b) discover ‘significant jumps’ as well as ‘co-jumps’ in most of the 12 cryptocurrencies studied using AR-GJR-GARCH models.

Bouri et al. (2021a) scrutinize the connectedness between seven cryptocurrencies using a quantile VAR methodology. They find the prominence of the conditional distributions’ tails, rather than their means or medians, in determining connectedness. Bouri et al. (2021b) find trading volume and uncertainties as key determinants of market during the increased integration of 12 top cryptocurrencies. Shahzad et al. (2021) analyze how COVID-19 affects the volatility spillover regimes of the daily returns of 18 important cryptocurrencies. High volatility regimes are found to transmit greater spillovers following the onset of the pandemic. Wątorek et al. (2021) show that cross-correlations between BTC/ETH–BTC/EUR and BTC/ETH–BTC/USD exchange rate pairs are characterized by a negative difference.

## Data and methodology

### Data and descriptive statistics

We use the intraday price data of six cryptocurrencies—Bitcoin (BTC), Ethereum (ETH), Litecoin (LTC), DASH, Ripple (XRP), and Monero (XMR)—at 15-min intervals. These assets represent the leading cryptocurrency in the market. The market capitalization of Bitcoin, Ethereum, Litecoin, and Ripple represents more than 80% of the total market capitalization of all cryptocurrencies (Naeem et al. 2021). The selected cryptocurrency assets, and especially Bitcoin, attract the attention of investors due to its innovative Blockchain technology and the significant opportunity to generate abnormal returns (Urqhuhart 2018; Yarovaya et al. 2021). The sample period ranges from September 1, 2017 to June 24, 2018. We have selected the same sample period for all cryptocurrencies to ensure a uniform analysis. This period has been marked by high levels of volatility and sharp fluctuations in cryptocurrency prices, as well heightened interest from investors (Bouri et al. 2020a). Data comes from the digital asset store Kaiko which offers tick-by-tick trade data for 6000+ currency pairs across 32+ exchanges including Bitfinex, the world’s leading cryptocurrency trading platform. We examine dollar-denominated data from Bitfinex exchange for our sample cryptocurrencies stamped at GMT time zone.

**Fig. 1:**
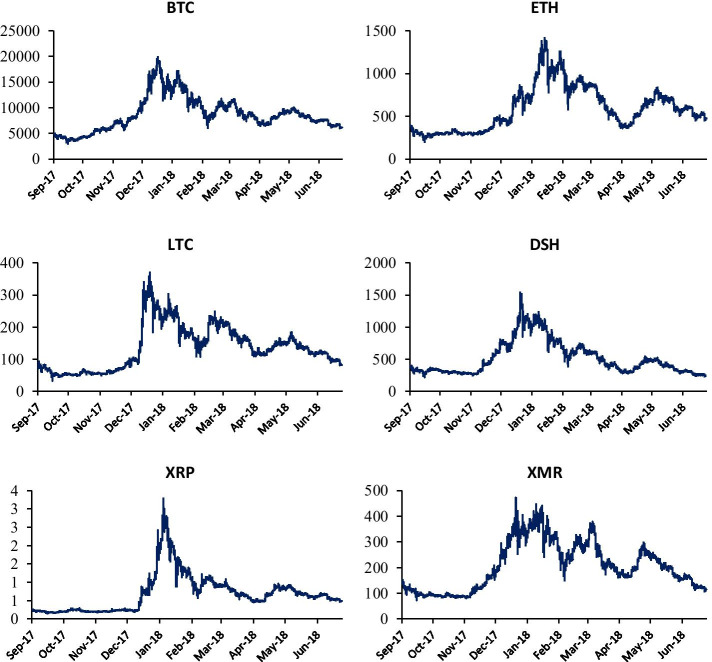
Evolution of 15-min prices of major cryptocurrencies

**Fig. 2:**
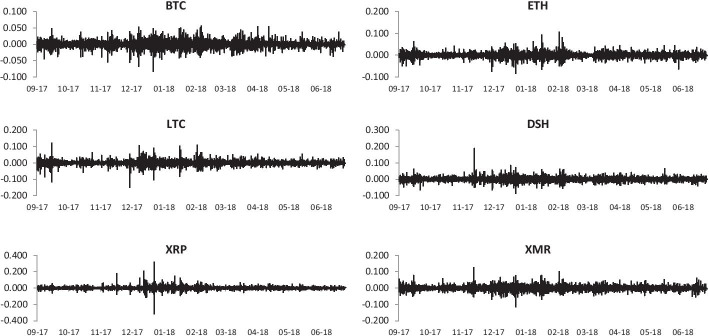
Time variations of 15-min price returns

Figure 1 plots the evolution of cryptocurrency prices and exhibits an upside trend for the period September 2017 to December 2017 (except Ripple). A significant structural break point is observed from early 2018 followed by a downside price trend. The price return series for all cryptocurrencies shows volatility clustering—especially between late 2017 and early 2018—and fat tails (Fig. 2). Table 1 shows that all cryptocurrency returns series are positive except DASH. Monero is the highest volatile market whereas BTC is the least one. Cryptocurrency price returns are characterized by heavy tails and volatility clustering related to nonlinear temporal correlations in the returns. This result is consistent with Wątorek et al. (2021). All returns series exhibit evidence against Gaussian distribution. The unit root tests of ADF and PP as well the stationary test of KPSS show that all cryptocurrency return series are stationary. The results of BDS test of Brock et al. (1996) show strong evidence of non-linearity across different embedding dimensions of the BDS test. This result indicates that the traditional linear models are not suitable to identify and capture the true nature of relationship among cryptocurrency returns.^3^

**Table 1:** Descriptive statistics

Variables	BTC	ETH	LTC	DASH	RIPPLE	MONERO
Mean	0.00001	0.00001	0.00000	− 0.00002	0.00000	0.00002
Maximum	0.05600	0.10600	0.12000	0.18800	0.12300	0.31400
Minimum	− 0.08200	− 0.08300	− 0.14800	− 0.08600	− 0.11300	− 0.31500
Std. Dev	0.00663	0.00723	0.00868	0.00773	0.00862	0.01067
Skewness	− 0.13686	0.04133	0.10145	0.76115	0.04843	0.75986
Kurtosis	12.22892	15.49612	21.22038	25.46886	14.54152	81.53078
Jarque–Bera	100,934*	184,893*	393,116*	600,486*	157,728*	7,304,562*
ADF	− 220.4327*	− 127.2089*	− 130.6018*	− 137.5965*	− 135.6117*	− 135.6117*
PP	− 315.2593*	− 292.4937*	− 298.2076*	− 290.0422*	− 304.6763*	− 304.6763*
KPSS	0.3022	0.1721	0.2496	0.2293	0.2344	0.2344

This table presents the descriptive statistics of 15-min prices of major cryptocurrencies for the period from September 1, 2017 to June 24, 2018

*Represents significance level at 5% or better

### Methodology

#### Wavelet correlation

In order to analyze the relationship between our sampled cryptocurrencies, we apply maximal overlap discrete wavelet transform (MODWT)^4^ of Gencay et al. (2002). The multi-resolution analysis (MRA) scale levels provide eight decomposition: D1 (15–30 min), D2 (30–60 min), D3 (60–120 min), D4 (120–240 min), D5 (240–480 min), D6 (480–960 min), D7 (960–1920 min) and D8 (1920–3840 min).

The expression for unbiased wavelet correlation for scale ${\uplambda }_{j}$ between X and Y is as follows:

1 $${\stackrel{\sim }{\rho }}_{XY}= \frac{cov ({\tilde{W }}_{Y,jt }, {\tilde{W }}_{Y,jt })}{\sqrt{var\{{\tilde{W }}_{X,jt }\}}var\{{\tilde{W }}_{X,jt }\}}= \frac{{\stackrel{\sim }{\gamma }}_{XY}({\lambda }_{j})}{{\stackrel{\sim }{\sigma }}_{X}^{2}({\lambda }_{j}){\stackrel{\sim }{\sigma }}_{Y}^{2}({\lambda }_{j})}$$

where ${\stackrel{\sim }{\gamma }}_{XY}({\lambda }_{j})$ represents unbiased wavelet covariance estimators between ${\tilde{W }}_{Y,jt}$ and ${\tilde{W }}_{Y,jt}$ (market coefficients). ${\stackrel{\sim }{\sigma }}_{X}^{2}\left({\lambda }_{j}\right)$ and ${\stackrel{\sim }{\sigma }}_{Y}^{2}({\lambda }_{j})$ are the unbiased wavelet variance estimators for X and Y, respectively with the associated scale ${\lambda }_{j}$. Based on MODWT, we define estimators of the unbiased wavelets as

2 $${\stackrel{\sim }{\sigma }}_{X}^{2}\left({\lambda }_{j}\right)= \frac{1}{{\tilde{N }}_{j}} \sum_{t={L}_{j-1}}^{N-1}{\tilde{W }}_{j,t}^{2}$$

In Eq. (2), ${\tilde{W }}_{j,t}^{2}$ represents *j*^th^ level of MODWT coefficient for X, ${L}_{j}=\left(2j-1\right)\left(L-1\right)+1$ represents length of ${\lambda }_{j}$ scale i.e. wavelet filter whereas $\tilde{N }=N- {L}_{j}+1$ is the number of coefficients that remain ineffective by the boundary. We follow the work of Whitcher et al. (1999)^5^ in constructing the confidence interval 100(1 − 2p)% for the wavelet coherence. The confidence interval for the wavelet coherence is expressed as tanh{h[${\stackrel{\sim }{\rho }}_{XY}({\lambda }_{j})] \pm {\varnothing }^{-1} (i-p) / \sqrt{{N}_{j}-3}\}$, where ${\varnothing }^{-1}$(p) highlights 100p% for standard normal distribution and h(${\stackrel{\sim }{\rho }}_{XY})=tan{h}^{-1}({\stackrel{\sim }{\rho }}_{XY})$ is the Fisher Z-transformation (see Gencay et al. 2002 for more details).

#### Rolling window wavelet correlation (RWWC)

To highlight the presence of temporal variations in wavelet correlation, we use rolling window wavelet correlation (RWWC) as a dynamic measure. In our study, we analyzed this rolling correlation as a visualized decomposed correlation, proposed by Polanco-Martínez and Abadie (2016), under time–frequency space. Since the work by Ranta (2010), this technique has been employed in several studies (for example, Dajcman et al. 2012; Benhmad 2013) because it allows for analysis under different time intervals. Following Ranta (2010), Dajcman et al. (2012) and Benhmad (2013), we compute pairwise RWWC by rolling forward a single data point centered around a time. Specifically, we estimate the RWWC using rolling window of 250 observations i.e. 250 observations of 15-min cryptocurrency pricing data (250 * 15 = 3750 min or 62.5 h). The time scale comprises 4 total wavelet scales out of which D1 and D2 represent more volatile events at higher frequency (short-run) whereas D3 and D4 are associated with changes at lower frequencies (long-run). We restrict the number of wavelets up to J = 8; however, we analyze only the first four scales.^6^

#### Nonlinear Granger causality

Diks and Panchenko (2006) proposed a nonlinear Granger causality test under a non-parametric framework to avoid the over-rejection problem of Hiemstra and Jones (1994).

The null hypothesis of Granger causality between the two series X_t_ and Y_t_ is based on the fact that X_t_ contains no information regarding Y_t+1_. We present two delay vectors ${X}_{t}^{lx}=({X}_{t-1 X+1,\dots .Xt})$ and ${Y}_{t}^{ly}=({Y}_{t-1 Y+1,\dots .Yt})$ where ${l}_{x}$, ${l}_{y}$ ≥ 1 highlight delays for ${X}_{t}$ and ${Y}_{t}$, respectively. We present the expression for the null hypothesis as

3 $${H}_{0}: {Y}_{t+1}\left|{(X}_{t}^{lx}{;Y}_{t}^{ly})\sim {Y}_{t+1}\right|{Y}_{t}^{ly}$$

We consider Z_t_ = Y_t_ + 1 as a null hypothesis and drop the time indices in Eq. (3). According to Bekiros and Diks (2008), the conditional distribution of Z given (X, Y) = (x, y) is similar to Z given Y = y. The null hypothesis of Eq. (3) is expressed as a joint distribution function under the joint probability density function f_X,Y,Z_ (x, y, z) with the associated marginal satisfying the following conditions:

4 $$\frac{{f}_{x,y,z}(x,y,z)}{{f}_{y}(y)}= \frac{{f}_{X,Y}(x,y)}{{f}_{y}(y)} \frac{{f}_{Y,Z}(y,z)}{{f}_{Y}(y)}$$

From the above equation, it is evident that X and Y are conditionally independent of Y = y for each value of fixed y (Diks and Panchenko 2006). We express the null hypothesis of Eq. (3) as

5 $$\mathrm{q}\equiv {\mathbb{E}} \left[{f}_{x,y,z}\left(X,Y,Z\right){f}_{y }\left(Y\right)-{f}_{X,Y}\left(X,Y\right){f}_{Y,Z}\left(Y,Z\right)\right]=0$$

In Eq. (5), ${\mathbb{E}}$ denotes the expectation operator, and the estimator for q according to Diks and Panchenko (2006) is expressed as

6 $${T}_{n}{(\varepsilon }_{n})=\frac{{(2\epsilon )}^{-{d}_{X}-{2d}_{Y}-{d}_{Z}}}{n(n-1)(n-2)}\sum_{i}\left[\sum_{k,k\ne 1}\sum_{j,j\ne 1}{I}_{ik}^{XYZ}{I}_{ij}^{Y}{I}_{ik}^{XY}{I}_{ij}^{YZ}\right]$$

The expression denotes ${I}_{ij}^{W}$ representing $(||{W}_{i}{W}_{j}||$< ε), where I is the indicator or characteristic function. ${W}_{i}$ and ${W}_{j}$ denote elements of ${d}_{w}$-variate random vector W. ε is the bandwidth whereas n represents sampling size. Considering the local density estimator of ${d}_{w}$-variate random vector W, which is expressed as ${\widehat{f}}_{W}{W}_{i}={2\in }^{{-d}_{W}}{(n-1)}^{-1}\sum_{j,j\ne 1}{I}_{ij}^{W}$, the T statistics is defined as

7 $${T}_{n}{(\varepsilon }_{n})=\frac{(n-1)}{n(n-2)}\sum_{i}\left[ {\widehat{f}}_{X,Y,Z}{(X}_{i}{Y}_{i}{Z}_{i}) {\widehat{f}}_{Y}({Y}_{i})- {\widehat{f}}_{X,Y}({X}_{i}{,Y}_{i}){\widehat{f}}_{Y,Z}({Y}_{i},{Z}_{i})\right]$$

In the above equation, ${\varepsilon }_{n}$ = ${C}_{n}^{-\beta },$ with β $\in$ (1/4, 1/3) and C > 0, and for the lag –1, i.e. ${l}_{x}= {l}_{y}= -1$, the T value consists of asymptotic normal distribution satisfying the below mentioned condition:

8 $$\sqrt{n}\frac{{(T}_{n}({\varepsilon }_{n})-q)}{{S}_{n}} \stackrel{d}{\to } N(\mathrm{0,1})$$

In Eq. (8), $\stackrel{d}{\to }$ represents convergence in distribution whereas ${S}_{n}$ represents asymptotic variance estimator, ${T}_{n}$ (see Bekiros and Diks 2008).

#### Hedging ratios and hedging effectiveness measure

In order to provide implications for investment in cryptocurrencies for hedging purpose, we examine whether Bitcoin along with other cryptocurrencies can minimize portfolio risk without lowering the expected returns. For this purpose, we consider Bitcoin with other cryptocurrencies as a hedged portfolio aiming to hedge exposure to cryptocurrencies price movements. For this purpose, we use a Dynamic Conditional Correlation Generalized Autoregressive Conditional (DCC-GARCH) model of Engle (2002) which possesses no asymmetric properties and is feasible to measure the hedging ratios, optimal weights in a portfolio and hedging effectiveness. Our aim is to construct a portfolio having minimum risk for expected returns. We follow the work by Kroner and Ng (1998) for estimating optimal weights of the Bitcoin in portfolio $\left({w}_{t}^{BTC}\right)$ at time $t$ as.

9 $${w}_{t}^{BTC}= \frac{{h}_{t}^{crypto}-{h}_{t}^{BTC,crypto}}{{h}_{t}^{BTC}-2{h}_{t}^{BTC,crypto}+{h}_{t}^{crypto}},\,\, with\,\, {w}_{t}^{BTC}=\left\{\begin{array}{ll}0& {w}_{t}^{BTC}<0\\ {w}_{t}^{crypto}& 0\le {w}_{t}^{BTC}\le 1\\ 1& {w}_{t}^{BTC}>1\end{array}\right.$$

where ${(h}_{t}^{crypto}), \left({h}_{t}^{BTC}\right)\,\, \mathrm{and}\,\, \left({h}_{t}^{BTC, crypto}\right)$ represents conditional volatility of cryptocurrencies other than the Bitcoin, conditional volatility of bitcoin asset and conditional covariance between Bitcoin and other cryptocurrencies asset, respectively. Expression for the optimal budget weight for cryptocurrencies other than the Bitcoin is presented as $({1-w}_{t}^{WTI}$).

For hedging ratios to minimize risk, we follow the work by Kroner and Sultan (1993) for constructing portfolio comprising of bitcoin and other cryptocurrencies (BTC and crypto). In order to minimize portfolio risk which is $1 long in cryptocurrencies contract, an investor should short $$$\beta$$ of the cryptocurrencies other than the Bitcoin. More specifically, hedging ratio with minimum variance at time *t* is appended below.

10 $${\beta }_{t}= \frac{{h}_{t}^{crypto-BTC}}{{h}_{t}^{BTC}}$$

Finally, we estimate hedging effectiveness ratio as highlighted below.

11 $$\mathrm{HE}=1-\frac{{\mathrm{Var}}_{\mathrm{hedged}}}{{\mathrm{Var}}_{\mathrm{unhedged}}},$$

where $Var_{hedged}$ and $Var_{unhedged}$ represents variance of the hedged portfolio (comprising of bitcoin and other cryptocurrencies) and unhedged portfolio (cryptocurrencies other than the bitcoin). Higher value of the HE ratio highlights higher hedging effectiveness.

## Results

### RWWC analysis

**Fig. 3:**
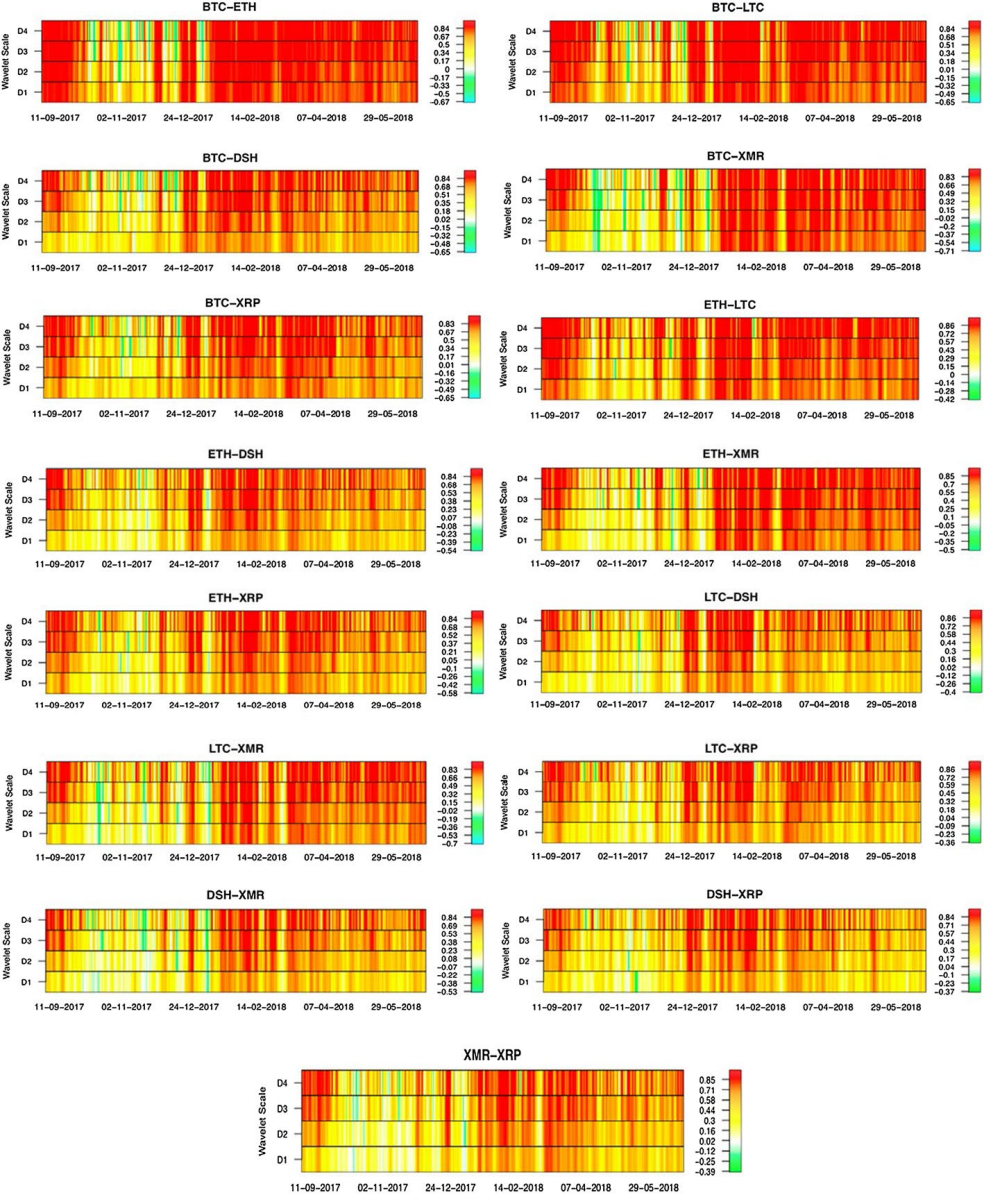
Rolling window wavelet correlation

The estimated RWCC coefficients—exhibited in Fig. 3—are, for the most part, all found to be non-zero. The lowest coefficient value reaches − 0.71 for BTC-XMR pair and the highest attains 0.86 for LTC-DSH, LTC-XRP and ETH-LTC pairs. The pairwise rolling window correlations for BTC-ETH range between values as low as − 0.5 to more than 0.8. Most of the lower correlation coefficients occurred between October and December 2017 and in all wavelet scales: D1 to D4 (from 0 to 120 min). This may be due to the upside trend of Bitcoin and Ethereum prices and an uptick in their volatility—observations that are shared by Corbet et al. (2018a,^ 2019^)—which are also observed in Figs. 1 and 2. There were also short bouts of low correlation in early- and mid-2018, mostly in D1 (15–30 min), D2 (30–60 min), and D4 (120–240 min) scales. The pattern of time-variant correlation coefficients is quite similar for the cryptocurrency pair BTC-LTC. The lower correlations occur in all wavelet scales between October and December 2017. However, there is a longer prevalence of lower wavelet correlations between early- and mid-2018 in scales D1, D2, and D4 of the BTC-LTC pair.

A recurring pattern of rolling window correlations can be seen in the cryptocurrency pairs BTC-DSH, BTC-XMR, and BTC-XRP. The correlation coefficients range between 0.8 and − 0.65, and the lower correlations including negative ones occur predominantly in the lower wavelet scales D1 and D2. In contrast, the higher wavelet scales are dominated by higher correlation coefficients. In particular, the lower correlations dominate the final three months of 2017 in all wavelet scales. For higher wavelet scales (such as D3 and D4), the lower correlation coefficients occur briefly, and intermittently, in early- and mid-2018. The lower correlations are also more prevalent in these three cryptocurrency pairs in contrast to BTC-ETH and BTC-LTC.

The cryptocurrency pairs involving Ethereum—ETH-LTC, ETH-DSH, ETH-XMR, and ETH-XRP—exhibit similar patterns. The rolling window wavelet correlation coefficients for these four pairs range between 0.85 and − 0.5. There are higher concentrations of lower correlations for the smaller wavelet scales (such as D1 and D2), and in the last quarter of 2017. This result reveals the possibility of diversification benefits—in line with Corbet et al. (2018a,^ 2019^) and Aslanidis et al. (2019). The lower correlations also occur sporadically, for the most part, in early- and mid-2018. However, there is a higher prevalence of the lower correlations in the cryptocurrency pairs ETH-DSH and ETH-XRP. It is worth noting that the lower correlation coefficients are positive for the most part, with only traces of negative correlations in the final quarter of 2017. The RWCC coefficients are higher in the bigger wavelet scales (e.g. D3–D4) for the pairs ETH-LTC and ETH-XMR. In addition, each of these two pairs of cryptocurrencies indicates a higher level of similarity among themselves (i.e. ETH-LTC is more akin to ETH-XMR than to the ETH-DSH or ETH-XRP pairs). The above observations are novel but somewhat akin to the observation of positive correlations between cryptocurrencies by Aslanidis et al. (2019) and that of clustering by Hu et al. (2019).

Similar patterns of wavelet correlations also exist between the LTC-DSH, LTC-XMR and LTC-XRP pairs. Lastly, we observe the rolling window wavelet correlation coefficients for cryptocurrency pairs DSH-XMR, DSH-XRP, and XMR-XRP. These cryptocurrency pairs also exhibit similar correlation patterns with lower correlation coefficients dominating the lower wavelet scales (D1 and D2) as well as the last quarter of 2017. The higher correlations dominate the higher wavelet scales (D3 and D4) and for much of early- to mid-2018. However, these cryptocurrency pairs also exhibit an increased prevalence of lower correlation coefficients in contrast to the cryptocurrency pairs involving Bitcoin and Ethereum.

The rolling window wavelet correlation coefficients reveal a general pattern: the correlations of cryptocurrencies are time-varying and more (positively) correlated in relatively higher wavelet scale; a pattern that appears in much of 2018 as well. There are also brief intermittent periods of low (including negative) correlations in 2018 as well as a long spell in the final quarter of 2017. High volatility and a rapid decline—possible indication of a bubble—is a characteristic of this period in the cryptocurrency marketplace (Corbet et al. 2018b,^ 2019^; Su et al. 2018). As such, we can affirm that the cryptocurrencies move together closely for most of the sample time period. In addition, long-term co-movements are relatively stronger than short-term ones—roughly similar to the evidence of jumping behavior amongst cryptocurrencies by Bouri et al. (2020b). This is in line with the extant literature such as Caporale et al. (2018), Phillip et al. (2019) and Qureshi et al. (2020), who detected long-term memory (persistence) in the cryptocurrency market. However, our analysis reveals that the long memory (persistence) is more transient in the lower wavelet scales than in the higher wavelet scales. This provides a unique time–frequency perspective to the memory characteristics of the cryptocurrency market—the cryptocurrencies co-move more closely over a longer span of time than over shorter time-spans. This implies that the cryptocurrencies move in the same direction over time despite the short-term deviations, which are likely due to the unique conditions of the particular cryptocurrency marketplace as well as the cryptocurrency’s technical specifications. These findings, although novel, are in agreement with Aslanidis et al. (2019), Bouri et al. (2020b), Rehman (2020), Bouri et al. (2021b), inter alia.

Moreover, the correlations appear to be higher for cryptocurrency pairs involving BTC and ETH, and to a lesser extent XMR. This may also be an indication of volatility connectedness (contagion) as well as strategic behavior and bargaining in this market (Hu et al. 2019).

### Nonlinear multiscale Granger causality analysis

**Fig. 4:**
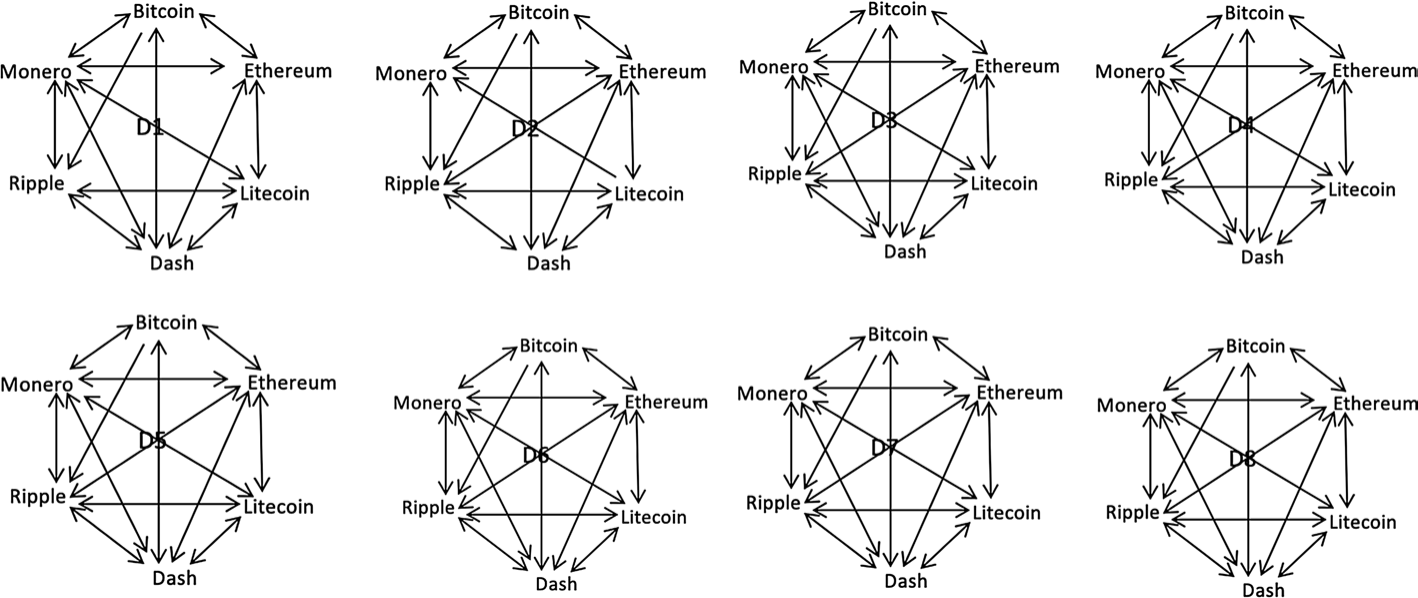
Non-linear causality at different MODWT levels (from D1 to D8). *Notes*: The arrows in the solid lines indicate the causality direction between each cryptocurrency market pair. The nonlinear causality between BTC and Litecoin is insignificant for this reason we did not have arrow

Table 3 provides the results of the nonlinear multiscale Granger causality test performed on the eight wavelet decomposed datasets. The estimated test statistics and associated *p*-values are given in Table 4. A visual representation of the Granger causality directions is provided for each MODWT level in Fig. 4. As can be seen, there is evidence of bidirectional Granger causation between each cryptocurrency pair for all wavelet scales, except for the following instances: non-causation between ETH-RIPPLE in D1 and well as between DASH and MONERO in D2, indicating evidence of hedging. We also find evidence of unidirectional causality from LTC to MONERO in D2, suggesting that LTC can be used to predict the MONERO price returns. The bidirectional nonlinear information spillover between each cryptocurrency pair reiterates the connectedness of the market and the substantial information spillover between cryptocurrencies as observed through the rolling window wavelet correlation analysis. In addition, causality estimates also reaffirm long-term memory across the cryptocurrencies. Thus, our findings add to earlier observations by Phillip et al. (2018, 2019), Bouri et al. (2020a) and Shahzad et al. (2021).

The nonlinear Granger causality results also reveal that ETH and MONERO—found previously (Fig. 3) to be more volatility connected than the rest of cryptocurrencies—appear to not impart volatility (information) to three other cryptocurrencies in the lower wavelet scales (i.e. in the short-term). This is a contrast to the findings of the rolling window wavelet correlation analysis. While Ethereum and Monero may volatility connected more than the others, they are less able to initiate short-term information spillovers—i.e. volatility spillover from them are sluggish compared to the others.

For robustness, we also apply the Diks and Panchenko (2006) Granger causality using multiple embedding dimensions to increase the robustness against the lag order. The results show non-linear bidirectional causality from Bitcoin to other cryptocurrencies and vice versa in all cases with consistency across all embedding dimensions. Our results imply more contemporary handling the asymmetric and non-linear properties of data. We also carry out the Markov regime switching model for two different regime classifications. The results show that in both regimes, though the coefficient remains significant, the variation in magnitude is quite high. The magnitude in the low volatility regime produces high variations in other cryptocurrencies whereas in the case of high volatility regime, the explanatory power of Bitcoin in other cryptocurrencies reduces significantly. Such asymmetric shift in the magnitude of returns across two regimes justifies our application of non-linear wavelets decomposed into different frequencies ranging from short- to long-run.^7^

### Portfolio analysis

To help investors to take optimal portfolio allocation decisions, we study the optimal portfolio design that includes BTC with another cryptocurrency asset (Dash, ETH, LTC, MNR, and XRP). Our assumption is that cryptocurrency investors may invest in another currency to hedge their position against downward BTC prices. This assumption can be justified by the increasing independence (i.e., ‘self-contained trade’) of the cryptocurrency markets observed by Drożdż et al. (2020). Table 2 provides a detailed portfolio risk assessment under different scales for designing optimal hedging strategies. The results of optimal weights show that investors should hold less BTC than other cryptocurrency asset (except for ETH for raw series, D1 and D6) in order to minimize the risk without lowering the expected return of BTC and each other cryptocurrency. This result persists regardless the frequencies. The average weight for BTC/ETH portfolio under scale D2 (30–60 min) is 0.294, indicating that for a US$10 on average 294 cents should be invested in ETH and the remaining budget 706 cents should be invested in BTC. The hedge ratio values oscillate between negative to positive values and are sensitive to the evolving of scales and the portfolio considered. For BTC/ETH portfolio under D2, a hedge ratio of 0.398 indicates that a US$ 1 long (buy) in the BTC requires investors to go short $0.398 in the ETH. The negative values of hedge ratios are observed for few cases, indicating that that the two crypto assets (i and j) are moving in opposite direction (negative correlation) in the short run. The hedging effectiveness results reveal that, in almost all cases, a mixed portfolio offers better risk reductions than individual BTC portfolio regardless of the scales and portfolio. Overall, the optimal weights, hedge ratios, and hedging effectiveness are sensitive to the time scale.

**Table 2:** Optimal weights, hedge ratios and hedging effectiveness under different scales

Portfolios	${W}_{t}^{BTC}$	${\beta }_{t}$	*RE*_*VAR*_ (%)
*Raw series*			
BTC/ETH	0.6332	0.8231	0.2899
BTC/LTC	− 0.0497	0.9119	0.3813
BTC/DASH	0.3435	0.6850	0.5135
BTC/XRP	0.1463	0.8543	0.4877
BTC/XMR	0.2196	0.8291	0.5359
*D1*			
BTC/ETH	2.3039	− 0.5336	− 2.6977
BTC/LTC	0.2102	1.4016	0.6771
BTC/DASH	0.4992	− 1.9546	0.9105
BTC/XRP	0.6801	− 1.2410	2.9236
BTC/XMR	0.2035	− 4.1168	1.1959
*D2*			
BTC/ETH	0.2939	0.3984	0.8973
BTC/LTC	0.3702	− 0.5863	1.3051
BTC/DASH	0.5371	1.0930	0.9968
BTC/XRP	− 0.1191	− 7.3638	0.7519
BTC/XMR	0.0948	− 8.2036	0.1589
*D3*			
BTC/ETH	− 0.3691	3.0295	0.7858
BTC/LTC	0.5080	1.0737	1.2410
BTC/DASH	0.0280	0.6639	0.8915
BTC/XRP	1.2612	3.4945	0.4041
BTC/XMR	− 0.1838	0.3284	1.5576
*D4*			
BTC/ETH	− 7.9228	− 0.0164	− 10.6329
BTC/LTC	− 0.0345	0.8847	1.3392
BTC/DASH	0.2852	0.5123	0.9385
BTC/XRP	− 0.2056	1.1843	0.7564
BTC/XMR	0.5518	2.4997	1.6471
*D5*			
BTC/ETH	− 1.0418	0.5409	2.2105
BTC/LTC	0.3046	0.7648	0.8204
BTC/DASH	− 0.2126	0.5997	0.7013
BTC/XRP	0.1156	0.6702	0.6843
BTC/XMR	0.6114	1.0588	0.8084
*D6*			
BTC/ETH	1.0857	0.5962	1.3132
BTC/LTC	0.1880	0.8720	0.4522
BTC/DASH	0.6469	0.3812	0.7056
BTC/XRP	− 0.0583	0.6003	0.6428
BTC/XMR	0.1302	0.8099	1.2568
*D7*			
BTC/ETH	− 0.8670	0.7372	0.5418
BTC/LTC	0.1298	0.7119	0.3139
BTC/DASH	0.0481	0.4515	0.6513
BTC/XRP	0.1935	− 0.0566	0.8699
BTC/XMR	4.2093	0.4772	93.0881
*D8*			
BTC/ETH	− 1.0611	0.8583	0.4838
BTC/LTC	0.0569	0.8583	− 0.5250
BTC/DASH	0.8044	0.0311	0.8686
BTC/XRP	0.6123	0.5866	0.5871
BTC/XMR	− 0.1864	0.1465	0.8040

In this table, we used dynamic conditional covariance between cryptocurrency returns along with their individual conditional variances using DCC model. The dynamic ratio is estimated using covariance between two cryptocurrency assets divided by variance of the benchmark BTC asset

## Conclusion

This study estimates rolling window wavelet correlation coefficients and nonlinear multiscale Granger causality tests to ascertain the time–frequency relationships across six prominent cryptocurrencies. Empirical wavelet correlation results indicate predominantly positive co-movements between the cryptocurrencies, especially between Bitcoin, Ethereum, and Monero. The nonlinear Granger causality tests reveal dual causation between most of the cryptocurrency pairs. These findings point to interconnectedness in the cryptocurrency marketplace.

Overall, both the RWCC and multiscale Granger causality analysis exhibit bidirectional correlations among cryptocurrencies and indicate that substantial opportunity for portfolio diversification (Das et al. 2018; Corbet et al. 2018b; Makarov and Schoar 2019). Our findings are augmented by robustness tests including nonlinear Granger causality of Diks and Panchenko (2006) and the Markov regime switching model. The results show non-linear bidirectional causality from Bitcoin to other cryptocurrencies and vice versa. In addition, we find asymmetric shift in the magnitude of returns across two regimes. Optimal portfolio design is sensitive to scales. Furthermore, a mixed portfolio composed from BTC and other cryptocurrency asset offers a better hedging effectiveness.

These findings should motivate policy makers to explore improvements to oversight mechanisms in the cryptocurrency markets and encourage them to promote a more mature and robust exchange system via the development of relevant infrastructure and regulations (Corbet et al. 2019). Finally, the results can also lead pertinent agents towards improved hedging and portfolio risk assessment strategies.

Though our work examines the presence of correlation using a contemporary rolling window wavelet measure, our work can further be extended in future by examining the portfolio implications of all cryptocurrencies. Since our work employs wavelet correlation, this can be extended to include the wavelet VaR analysis and opinion dynamics (Zha et al. 2021) which can have important implications for investors holding cryptocurrencies portfolio.

## Data Availability

The datasets used and analyzed during the current study are available from the corresponding author on reasonable request.

## Appendix

### Wavelet decomposition

The advantage of using discrete wavelet transformation (DWT) is its ability to model non-stationary time series in a time-scale framework (Gencay et al. 2002). Maximal overlap discrete wavelet transformation (MODWT) differentiates itself from traditional DWT methodologies due to its ability to handle sample restrictions (i.e. size up to 2^J^, J = decomposition layers), making it one of the most commonly used algorithms (Percival and Walden 2006). Another advantage of the MODWT technique is its invariability to circular shifting of time series, not supported by traditional DWT. A final advantage of the DWT and MODWT models is that they have the ability to analyze variance based on wavelets and scaling coefficients, with the variance estimators being more asymptotically efficient.^8^

In this paper, we decompose the 15 min tick returns of cryptocurrencies using MODWT with Daubechies least asymmetric wavelets^9^ LA(8) (i.e. a filter of length L = 8). We use *log*_2_(*N*) for extracting maximum decomposition level J. Because the number of wavelet coefficients tends to become critically small at high levels, we select J = 8 for avoiding boundary coefficients. Therefore, by using MODWT under this arrangement, we get eight wavelets with one scaling coefficient (i.e. ${\stackrel{\sim }{\omega }}_{1,t}, \dots .,{\stackrel{\sim }{\omega }}_{8,t}$ and ${\tilde{v }}_{8,t}$, respectively). In the case of rolling window wavelet correlation (discussed earlier), we take J = 4. Although the possibility of selecting J = 5 exists, the analysis does not provide meaningful results. The required level of transformation defines the scale of wavelet coefficient, i.e. ${\stackrel{\sim }{\omega }}_{t,j}$. For all families of Daubechies, the level J has association with changes at the effective scale ${\uplambda }_{j}= {2}^{j-1}$ days. The MODWT also encompasses idea bandpass filters between the frequency intervals [${1/2}^{j+1},{1/2}^{j}]$ for scale 1 ≤ j ≤ J. To obtain equivalent periods of [${2}^{j},{2}^{j+1}]$∆t under 1 ≤ j ≤ J scale, we invert the frequency range and then multiply it by an appropriate time unit (the 15 min tick price in our case). In our study, the wavelet coefficients λj, j = 1, … 8 are associated with time horizon changes of 15, 30, 60, 120, 240, 480, 960, and 1920 min, respectively. We decompose original return series of 15-min frequency into eight different components. Such decomposition helps in examining the wavelet correlation based on specified rolling window across different frequencies. These frequencies are highlighted by the decomposed levels ranging from D1 to D8 corresponding with higher frequency (short-run) to low frequency (long-run). The reason to use 8 scales rather than 6 is to use maximum scales for more clear interpretation across different investment horizons. Eight scales present more frequency windows to analyze correlation between our sampled cryptocurrencies. Furthermore, we decompose default return series using MODWT which employs Daubechies least asymmetric (LA) wavelet filter of length = 8, commonly referred to as LA (8) (see Daubechies 1992; Gencay et al. 2002) (Tables 3, 4).

**Table 3:** Non-linear directional Granger causality

Cryptocurrency pair	MODWT level							
	D1	D2	D3	D4	D5	D6	D7	D8
BTC-ETH	↔	↔	↔	↔	↔	↔	↔	↔
BTC-LTC	↔	↔	↔	↔	↔	↔	↔	↔
BTC-DASH	↔	↔	↔	↔	↔	↔	↔	↔
BTC-RIPPLE	↔	↔	↔	↔	↔	↔	↔	↔
BTC-MONERO	↔	↔	↔	↔	↔	↔	↔	↔
ETH-LTC	↔	↔	↔	↔	↔	↔	↔	↔
ETH-DASH	↔	↔	↔	↔	↔	↔	↔	↔
ETH-RIPPLE	Insignificant	↔	↔	↔	↔	↔	↔	↔
ETH-MONERO	↔	↔	↔	↔	↔	↔	↔	↔
LTC-DASH	↔	↔	↔	↔	↔	↔	↔	↔
LTC-RIPPLE	↔	↔	↔	↔	↔	↔	↔	↔
LTC-MONERO	↔	→	↔	↔	↔	↔	↔	↔
DASH-RIPPLE	↔	↔	↔	↔	↔	↔	↔	↔
DASH-MONERO	↔	Insignificant	↔	↔	↔	↔	↔	↔
RIPPLE-MONERO	↔	↔	↔	↔	↔	↔	↔	↔

This table presents the results of pairwaise non-linear Granger causality test of Diks and Panchenko (2006). For each MODWT level (D1 to D8), the arrows → and ↔ stand respectively for statistically significant unidirectional and bidirectional relationship between cryptocurrency pairs

**Table 4:** Estimates for non-linear Granger causality at decomposed levels

Direction	MODWT level							
	D1	D2	D3	D4	D5	D6	D7	D8
BTC-ETH	15.181 (0.0000)	14.912 (0.0000)	19.798 (0.0000)	18.555 (0.0000)	13.525 (0.0000)	8.464 (0.0000)	7.260 (0.0000)	6.737 (0.0000)
ETH-BTC	16.992 (0.0000)	17.106 (0.0000)	21.198 (0.0000)	21.401 (0.0000)	16.181 (0.0000)	12.480 (0.0000)	10.756 (0.0000)	9.947 (0.0000)
BTC-LTC	18.199 (0.0000)	19.202 (0.0000)	23.273 (0.0000)	21.368 (0.0000)	14.241 (0.0000)	10.366 (0.0000)	8.515 (0.0000)	6.737 (0.0000)
LTC-BTC	19.511 (0.0000)	19.673 (0.0000)	22.123 (0.0000)	21.983 (0.0000)	17.462 (0.0000)	12.569 (0.0000)	12.523 (0.0000)	9.947 (0.0000)
BTC-DASH	21.474 (0.0000)	26.311 (0.0000)	25.656 (0.0000)	25.123 (0.0000)	17.783 (0.0000)	12.154 (0.0000)	9.556 (0.0000)	8.122 (0.0000)
DASH-BTC	20.131 (0.0000)	21.550 (0.0000)	20.157 (0.0000)	19.941 (0.0000)	16.106 (0.0000)	12.430 (0.0000)	10.485 (0.0000)	10.881 (0.0000)
BTC-RIPPLE	17.322 (0.0000)	18.120 (0.0000)	22.683 (0.0000)	21.229 (0.0000)	15.791 (0.0000)	11.148 (0.0000)	7.750 (0.0000)	8.616 (0.0000)
RIPPLE-BTC	17.868 (0.0000)	17.346 (0.0000)	17.623 (0.0000)	17.757 (0.0000)	16.074 (0.0000)	11.521 (0.0000)	11.380 (0.0000)	10.274 (0.0000)
BTC-MONERO	19.900 (0.0000)	27.997 (0.0000)	22.323 (0.0000)	21.654 (0.0000)	15.977 (0.0000)	11.206 (0.0000)	6.089 (0.0000)	8.074 (0.0000)
MONERO-BTC	20.034 (0.0000)	23.647 (0.0000)	20.899 (0.0000)	20.228 (0.0000)	15.346 (0.0000)	11.773 (0.0000)	13.057 (0.0000)	9.945 (0.0000)
ETH-LTC	20.622 (0.0000)	21.164 (0.0000)	24.751 (0.0000)	23.147 (0.0000)	16.791 (0.0000)	13.197 (0.0000)	10.629 (0.0000)	9.197 (0.0000)
LTC-ETH	19.379 (0.0000)	19.295 (0.0000)	21.919 (0.0000)	21.371 (0.0000)	15.793 (0.0000)	10.737 (0.0000)	10.635 (0.0000)	11.139 (0.0000)
ETH-DASH	18.046 (0.0000)	20.052 (0.0000)	23.368 (0.0000)	24.149 (0.0000)	18.389 (0.0000)	13.318 (0.0000)	11.645 (0.0000)	8.078 (0.0000)
DASH-ETH	17.273 (0.0000)	15.384 (0.0000)	17.179 (0.0000)	17.357 (0.0000)	13.211 (0.0000)	9.683 (0.0000)	8.403 (0.0000)	10.259 (0.0000)
ETH-RIPPLE	0.883 (0.1886)	23.647 (0.0000)	28.572 (0.0000)	24.716 (0.0000)	18.870 (0.0000)	13.987 (0.0000)	11.139 (0.0000)	11.390 (0.0000)
RIPPLE-ETH	0.883 (0.1886)	17.064 (0.0000)	18.066 (0.0000)	17.106 (0.0000)	14.736 (0.0000)	10.198 (0.0000)	9.783 (0.0000)	7.895 (0.0000)
ETH-MONERO	18.393 (0.0000)	18.684 (0.0000)	21.988 (0.0000)	21.699 (0.0000)	17.109 (0.0000)	11.754 (0.0000)	11.139 (0.0000)	6.236 (0.0000)
MONERO-ETH	17.563 (0.0000)	16.998 (0.0000)	19.655 (0.0000)	19.086 (0.0000)	13.431 (0.0000)	10.907 (0.0000)	9.783 (0.0000)	10.716 (0.0000)
LTC-DASH	20.454 (0.0000)	21.231 (0.0000)	23.638 (0.0000)	24.133 (0.0000)	18.811 (0.0000)	13.386 (0.0000)	11.891 (0.0000)	9.844 (0.0000)
DASH-LTC	19.138 (0.0000)	17.071 (0.0000)	19.334 (0.0000)	19.023 (0.0000)	13.242 (0.0000)	10.446 (0.0000)	8.905 (0.0000)	9.655 (0.0000)
LTC-RIPPLE	22.893 (0.0000)	20.023 (0.0000)	25.736 (0.0000)	22.774 (0.0000)	18.249 (0.0000)	12.621 (0.0000)	10.686 (0.0000)	8.558 (0.0000)
RIPPLE-LC	21.249 (0.0000)	19.974 (0.0000)	19.941 (0.0000)	19.788 (0.0000)	15.190 (0.0000)	11.282 (0.0000)	9.275 (0.0000)	8.181 (0.0000)
LTC-MONERO	20.759 (0.0000)	0.472 (0.0000)	22.838 (0.0000)	22.550 (0.0000)	17.938 (0.0000)	12.355 (0.0000)	8.701 (0.0000)	8.794 (0.0000)
MONERO-LTC	20.643 (0.0000)	0.472 (0.3186)	22.093 (0.0000)	20.783 (0.0000)	14.666 (0.0000)	11.374 (0.0000)	11.438 (0.0000)	9.821 (0.0000)
DASH-RIPPLE	17.735 (0.0000)	16.209 (0.0000)	18.569 (0.0000)	17.040 (0.0000)	13.824 (0.0000)	10.070 (0.0000)	10.059 (0.0000)	9.062 (0.0000)
RIPPLE-DASH	19.948 (0.0000)	19.754 (0.0000)	21.035 (0.0000)	21.429 (0.0000)	17.438 (0.0000)	13.046 (0.0000)	9.961 (0.0000)	9.056 (0.0000)
DASH-MONERO	19.877 (0.0000)	0.472 (0.3186)	20.245 (0.0000)	20.154 (0.0000)	15.236 (0.0000)	10.487 (0.0000)	7.521 (0.0000)	8.252 (0.0000)
MONERO-DASH	20.795 (0.0000)	0.472 (0.3186)	23.077 (0.0000)	23.035 (0.0000)	17.801 (0.0000)	12.883 (0.0000)	12.531 (0.0000)	10.754 (0.0000)
RIPPLE-MONERO	20.059 (0.0000)	19.586 (0.0000)	20.034 (0.0000)	19.658 (0.0000)	16.479 (0.0000)	11.202 (0.0000)	7.521 (0.0000)	7.387 (0.0000)
MONERO-RIPPLE	22.658 (0.0000)	22.320 (0.0000)	23.282 (0.0000)	20.831 (0.0000)	15.440 (0.0000)	11.335 (0.0000)	12.531 (0.0000)	9.994 (0.0000)

This table presents coefficients for non-linear Granger causality test for different decomposition levels ranging from D1 to D8. We highlight significance level as *p* values in parenthesis

## Abbreviations

DWT: Discrete wavelet transformation
MODWT: Maximal overlap discrete wavelet transformation
RWCC: Rolling window wavelet correlation
BTC: Bitcoin
ETH: Ethereum
LTC: Litecoin
XRP: Ripple
XMR: Monero

## Footnotes

1 Kou et al. (2021b) develop a bankruptcy prediction model for small and medium-sized enterprises (SMEs) that uses transactional data under a scenario where no accounting data are required.

2 Also see Rehman and Kang (2020).

3 The results of BDS test are available upon request.

4 For more information on wavelet decomposition analysis see “Appendix”.

5 Whitcher et al. (1999) explains the Fisher Z-transformation of correlation coefficient.

6 The reason for this is that after applying MODWT to a sub-window with 250 data points and avoiding boundary wavelet coefficients, data points become much less than 250 for the 5th scale. Computations are made as N–W, where N = 1043 and w = 250 resulting in N–W = 793 windows and therefore the resulting correlation coefficient.

7 The results of Granger causality test of Diks and Panchenko (2006) and the Markov regime switching model are available upon request.
